# Oxidative stress induces release of mitochondrial DNA into the extracellular space in human placental villous trophoblast BeWo cells

**DOI:** 10.1152/ajpcell.00091.2024

**Published:** 2024-05-13

**Authors:** Jennifer J. Gardner, Spencer C. Cushen, Reneé de Nazaré Oliveira da Silva, Jessica L. Bradshaw, Nataliia Hula, Isabelle K. Gorham, Selina M. Tucker, Zhengyang Zhou, Rebecca L. Cunningham, Nicole R. Phillips, Styliani Goulopoulou

**Affiliations:** ^1^Department of Physiology and Anatomy, University of North Texas Health Science Center, Fort Worth, Texas, United States; ^2^Texas College of Osteopathic Medicine, University of North Texas Health Science Center, Fort Worth, Texas, United States; ^3^Department of Microbiology, Immunology, and Genetics, University of North Texas Health Science Center, Fort Worth, Texas, United States; ^4^Department of Population & Community Health, University of North Texas Health Science Center, Fort Worth, Texas, United States; ^5^Department of Pharmaceutical Sciences, System College of Pharmacy, University of North Texas Health Science Center, Fort Worth, Texas, United States; ^6^Lawrence D. Longo, MD Center for Perinatal Biology, Departments of Basic Sciences, Gynecology, and Obstetrics, Loma Linda University School of Medicine, Loma Linda, California, United States

**Keywords:** mitochondrial DNA, placenta, pregnancy, reactive oxygen species, trophoblasts

## Abstract

Circulating cell-free mitochondrial DNA (ccf-mtDNA) is an indicator of cell death, inflammation, and oxidative stress. ccf-mtDNA in pregnancies with placental dysfunction differs from that in healthy pregnancies, and the direction of this difference depends on gestational age and method of mtDNA quantification. Reactive oxygen species (ROS) trigger release of mtDNA, yet it is unknown whether trophoblast cells release mtDNA in response to oxidative stress, a common feature of pregnancies with placental pathology. We hypothesized that oxidative stress would induce cell death and release of mtDNA from trophoblast cells. BeWo cells were treated with antimycin A (10–320 µM) or rotenone (0.2–50 µM) to induce oxidative stress. A multiplex real-time quantitative PCR (qPCR) assay was used to quantify mtDNA and nuclear DNA in membrane-bound, non-membrane-bound, and vesicle-bound forms in cell culture supernatants and cell lysates. Treatment with antimycin A increased ROS (*P* < 0.0001), induced cell necrosis (*P* = 0.0004) but not apoptosis (*P* = 0.6471), and was positively associated with release of membrane-bound and non-membrane-bound mtDNA (*P* < 0.0001). Antimycin A increased mtDNA content in exosome-like extracellular vesicles (vesicle-bound form; *P* = 0.0019) and reduced autophagy marker expression (LC3A/B, *P* = 0.0002; p62, *P* < 0.001). Rotenone treatment did not influence mtDNA release or cell death (*P* > 0.05). Oxidative stress induces release of mtDNA into the extracellular space and causes nonapoptotic cell death and a reduction in autophagy markers in BeWo cells, an established in vitro model of human trophoblast cells. Intersection between autophagy and necrosis may mediate the release of mtDNA from the placenta in pregnancies exposed to oxidative stress.

**NEW & NOTEWORTHY** This is the first study to test whether trophoblast cells release mitochondrial (mt)DNA in response to oxidative stress and to identify mechanisms of release and biological forms of mtDNA from this cellular type. This research identifies potential cellular mechanisms that can be used in future investigations to establish the source and biomarker potential of circulating mtDNA in preclinical experimental models and humans.

## Graphical abstract

**Figure:**
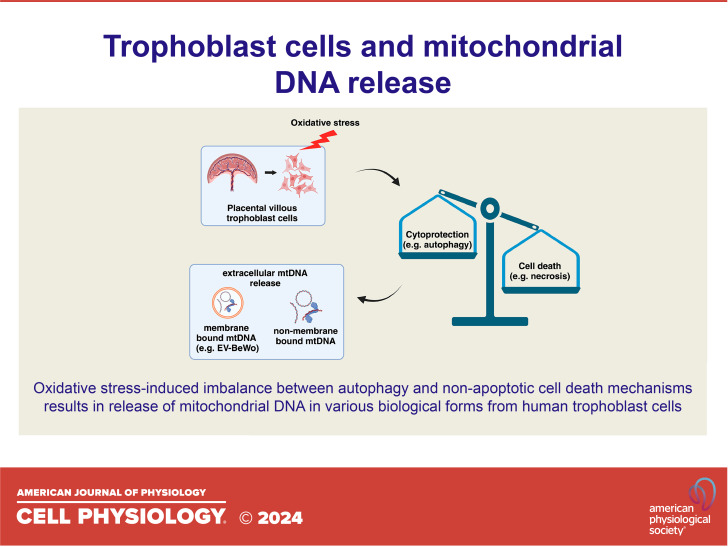

## INTRODUCTION

The placenta forms a critical interface between the mother and the developing fetus by functioning as the master regulator of fetal-maternal exchange of metabolic, inflammatory, and endocrine factors (1–3). The hemochorial orientation of the human placenta facilitates this communication by providing a limited cellular barrier between the maternal blood and fetal epithelium (4). This cellular barrier that forms the fetal-maternal interface is composed of trophoblast cells. In pathological conditions, trophoblast stress results in the release of proinflammatory and vasoactive factors that because of this hemochorial architecture can directly access the maternal vasculature, affecting its function and maternal outcomes during pregnancy (2).

Circulating cell-free mtDNA (ccf-mtDNA) is a damage-associated molecular pattern that is recognized by pattern recognition receptors of the innate immune system to elicit inflammation and facilitate immune system activation (5). ccf-mtDNA increases with advancing gestational age in maternal sera of healthy pregnant individuals (6), potentially mirroring placental and fetal developmental trajectories and trophoblast cell turnover. In preeclampsia, a severe hypertensive disorder of pregnancy with placental pathology, ccf-mtDNA concentrations differ from those in healthy control subjects. Some studies have reported an increase (7, 8) and others showed a reduction (9, 10) in maternal ccf-mtDNA in patients with preeclampsia, depending on gestational age and method of mtDNA quantification (e.g., relative vs. absolute quantification, primers used) (11).

In nonpregnant states, various types of cells have the ability to release mtDNA into circulation (12–14). In fact, damaged or dying nonplacental cells release mtDNA into the extracellular space during programmed cell death (i.e., apoptosis) or passive cell death (i.e., necrosis) (5, 11, 15). An increase in production of reactive oxygen species (ROS) in nonplacental tissues is a known trigger of mtDNA release (16, 17). In pregnancies with placental ischemia, a common feature of preeclampsia and a major contributor to adverse maternal and perinatal outcomes (2, 18), the placenta shows signs of necrosis, exaggerated apoptosis, and oxidative stress (2). Nevertheless, it remains unknown whether placental cells could also release mtDNA in response to activation of these mechanisms. Thus, the ability of placental cells to contribute to ccf-mtDNA and the mechanisms of placental release of extracellular mtDNA in response to stressors associated with pregnancy complications remain unknown.

ccf-mtDNA is transported in various biological forms (11). It can be fragmented or intact, naked, protein-associated, and encapsulated in vesicular structures or whole mitochondria (11). During pregnancy, most ccf-DNA is membrane bound (as compared to non-membrane-bound form), and this phenomenon is more pronounced in pregnancies with preeclampsia compared to healthy pregnancy (10). Many studies report ccf-mtDNA quantities without assessing the form of transport. Investigation of the form of transport of mtDNA may reveal information about the source and signaling potential of ccf-mtDNA.

The objective of this study was to elucidate the relationship between pathological oxidative stress, cell death, and mtDNA release in trophoblast cells, which are the primary cellular units of the maternal-fetal interface in the placenta. We hypothesized that trophoblast cells exposed to elevated levels of oxidative stress (i.e., reactive oxygen species, ROS) would release mtDNA into the extracellular space via cell death-dependent mechanisms. To test this hypothesis, we used a well-established in vitro model of human trophoblast cells (BeWo cells). Given that mtDNA is detected in the circulation in various biological forms (11), we also sought to determine biological forms of mtDNA released from trophoblast cells (i.e., membrane-bound, non-membrane-bound, and vesicle-bound mtDNA).

## MATERIALS AND METHODS

### Chemicals and Reagents

All chemicals and reagents are included in Supplemental Table S1 (see https://doi.org/10.6084/m9.figshare.25761720) unless otherwise indicated.

### Cell Culture

BeWo choriocarcinoma cells (sex: male; ATCC, catalog no. CCL-98, RRI:CVCL_0044) are a model of human epithelial cytotrophoblast cells (mononuclear) that form the progenitor trophoblast cells of the placenta. BeWo cells were grown in Kaighn’s modification of Ham’s F-12 medium (ATCC, catalog no. 30-2004), supplemented with 10% fetal bovine serum (FBS; ATCC, catalog. no. 30-2020) and 1% penicillin-streptomycin (Gibco, catalog no. 15140). The cells were maintained at 37°C in a >90% relative humidity incubator with 5% CO_2_ (HeraCell 150i, model 1335; ThermoFisher Scientific). Routine subculturing was performed with 0.25% trypsin-EDTA (Gibco, catalog no. 25200-056). BeWo cells were authenticated through short tandem repeat profiling (ATCC). To establish the “trophoblast” nature of the BeWo cell line, cells were stained for anti-cytokeratin 7 using immunocytochemistry [Supplemental Methods (see https://doi.org/10.6084/m9.figshare.25761732) and Supplemental Fig. S1 (see https://doi.org/10.6084/m9.figshare.25091609) (69).

### Experimental Design and Methods

**Figure 1.**
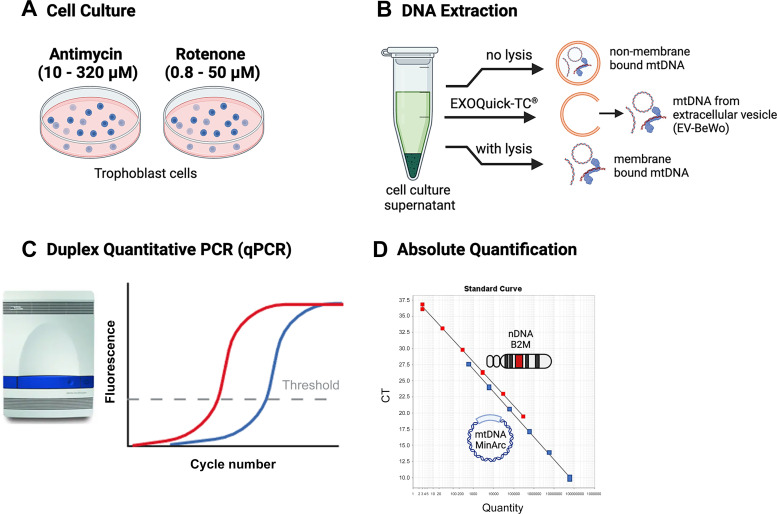
DNA extraction and quantification of mitochondrial (mt)DNA. *A*: BeWo cells were exposed to rotenone or antimycin A for 4 h. *B*: DNA was extracted from the supernatant of BeWo cells with lysis buffer (membrane-bound form), without lysis buffer (non-membrane-bound form), or with ExoQuick-TC method to isolate DNA stored within extravesicular membranes (EV-BeWo). *C* and *D*: mtDNA and nuclear (n)DNA were quantified with a duplex quantitative (q)PCR protocol (*C*) targeting the nuclear primer (B2M) and the mtDNA primer (MinArc), using TaqMan probes and chemistry (*D*). CT, cycle threshold. Created with BioRender.com.

After reaching 80% confluence, cells were treated for 4 h with antimycin A (10, 50, 100, 320 µM unless otherwise noted), or rotenone (0.2, 0.8, 5, 10, 25, 50 µM unless otherwise noted) or their respective vehicles [ethanol 0.5% (vol/vol) for antimycin A or DMSO 0.08% (vol/vol) for rotenone] (19). Real-time quantitative PCR (qPCR) with TaqMan probes and chemistry was used to quantify mtDNA and nuclear DNA in cell culture supernatants, cell lysates, and cell culture supernatant-derived exosome-like extracellular vesicles (EV-BeWo; Fig. 1). Oxidative stress was measured with a 2′,7′-dichlorofluorescein diacetate (DCFDA) assay. Flow cytometry was used to assess cell viability, apoptosis, and necrosis. Western blot analysis was used to assess protein expression of autophagy markers.

### Isolation and Characterization of Exosome-like Extracellular Vesicles

FBS was depleted of exosomes via ultracentrifugation (Thermo Scientific, Sorvall WX + Ultra Series) at 10,000 *g* for 2 h as previously described (20). The exosome-depleted FBS aqueous layer was then filtered through a 0.2-µm-sized 50-mL tube top vacuum filter system (catalog no. 430320, Corning, New York). Exosome-depleted medium (Kaighn’s modification of Ham’s F-12 medium supplemented with 10% exosome-depleted FBS and 1% penicillin-streptomycin) was used to seed BeWo cells into 100-mm petri dishes at a density of 100,000 cells/mL. The cells were incubated for ∼24 h at 37°C until ∼70% confluent. The cells were washed with phosphate-buffered saline (PBS) and then treated with antimycin A (320 µM) or vehicle for 4 h. Extracellular vesicles were isolated from 10 mL of cell culture supernatant with the ExoQuick-TC Exosome Precipitation Solution (catalog no. EXOTC10A-1, Systems Biosciences) according to the manufacturer’s instructions with a few modifications. Supernatants were collected and centrifuged at 3,000 *g* for 15 min and then again at 7,500 rpm for 10 min at 4°C to remove any cells and/or cellular debris. We added 1.5 times the recommended volume of ExoQuick-TC to 9.4 mL of clarified BeWo cell culture supernatant and incubated overnight. Extracellular vesicles were collected by centrifuging at 1,500 *g* for 30 min and removing the supernatant. This centrifugation step was repeated twice to ensure the complete removal of medium from the pellet. Extracellular vesicles were then resuspended in 250 µL of PBS and stored at −80°C until DNA extraction. Exosome-like extracellular vesicles from BeWo cells were designated as “EV-BeWo.”

Characterization of extracellular vesicles was performed by Systems Biosciences with an Exo-Check exosome antibody array (ExoRay200B, catalog no. CSEQ100A-1, Palo Alto, CA) and nanoparticle tracking analysis (NTA; Nanosight, version 2.3, Build 2.3.5.0033.7-Beta-7, catalog no. CSNANO100A-1). The array was exposed to 50 µg of exosome proteins isolated from supernatants of BeWo cells cultured in exosome-depleted media. The following exosome markers were used in the array: CD63 and CD81 (tetraspanins), EpCam (epithelial cell adhesion molecule; often found in cancer-derived exosomes), ANX5 (annexin 5), TSG01 (tumor susceptibility gene 101), FLOT-1 (flotillin-1), ICAM-1 (intercellular adhesion molecule 1), ALIX [programmed cell death 6 interacting protein (PDCD6IP)]. The NTA analysis with Systems Biosciences used a proprietary fluorescent dye that binds to intact membrane of extracellular vesicles, ExoGlow-NTA, to detect size and concentration of extracellular vesicles.

### DNA Extraction

DNA was measured in cell suspensions and cell culture supernatants from the same experiment. Cell culture supernatants were collected from the cell culture flask after 4-h exposure to rotenone, antimycin A, or vehicle. Adherent cells were collected with 0.25% trypsin-EDTA and centrifugation for 5 min at 400 *g* at 4°C. Cell pellets were resuspended in elution buffer. Independent experiments were performed to determine DNA content in extracellular vesicles.

#### Cell suspensions.

DNA was extracted from cell suspensions with the Mag-Bind Blood & Tissue DNA HDQ extraction kit (Omega Bio-Tek, Norcross, GA) according to manufacturer’s specifications in the Mag-Bind Blood protocol. DNA extracts were quantified with the Qubit dsDNA Broad Range assay kit (ThermoFisher Scientific) according to manufacturer’s recommendations. Samples with low concentrations were concentrated by spinning in the Vacufuge vacuum concentrator (Eppendorf) until concentrations reached at least 2.5 ng/µL, adequate for use in qPCR.

#### Cell culture supernatants.

DNA was extracted from supernatants twice with the Mag-Bind Blood & Tissue DNA HDQ extraction kit (Omega Bio-Tek) according to manufacturer’s specifications in the Mag-Bind Blood protocol with modifications. The first of the two DNA extractions was performed including a lysis step and the second extraction did not include the lysis step (10), to determine the contribution of membrane-bound and non-membrane-bound DNA, respectively.

#### Extracellular vesicles.

DNA was extracted from 200 µL of the resuspended extracellular vesicles with the DNeasy Blood & Tissue Kit (Qiagen, catalog no. 69504) according to the instructions in the Purification of Total DNA from Animal Blood or Cells (Spin-Column) Protocol. DNA samples were eluted with 200 µL of nuclease-free water instead of the elution buffer (Buffer AE) provided in the extraction kit. After DNA extraction, samples were concentrated by spinning in the Vacufuge vacuum concentrator (Eppendorf) until they reached a volume of 20 µL.

### DNA Quantification via Polymerase Chain Reaction

Multiplex quantification of nuclear DNA and mtDNA was performed according to Phillips et al. (21) and as we previously published (10), with some modifications. Namely, the deletion target was omitted. The master mix was modified as follows: 2 µL of each primer MinArc (0.625 µM) and B2M (7.5 µM); 1.25 µL of each probe MinArc (2 µM) and B2M (4 µM); 12.5 µL of TaqMan Universal MasterMix II, no UNG (Applied Biosystems, catalog no. 4440040); 2 µL of DNA extract. The concentration of DNA obtained from the supernatants was too low to be quantified, so it was used directly for qPCR without quantifying. DNA (5 ng) from cell suspension extracts was added to qPCR reaction mix. Standard curves based on known synthetic DNA copy number for both targets were used. *R*^2^ values and amplification efficiencies of the standard curves were analyzed for each plate to verify repeatability and monitor for potential batch effects. Negative controls were included on every run to monitor basal off-target mtDNA amplification.

### Cytosolic Oxidative Stress Measurements via DCFDA Assay

A DCFDA assay was performed according to manufacturer’s instructions (https://www.abcam.com/products/assay-kits/dcfda–h2dcfda-cellular-ros-assay-kit-ab113851.html; for details see Adherent cells protocol for microplate reader). Specifically, BeWo cells were seeded into a black-walled clear-bottom 96-well plate at a density of 25,000 cells/well and grown until 80% confluent. Cells were washed with 100 μL of PBS and stained with 25 μM DCFDA for 45 min in a cell culture incubator (37°C, 5% CO_2_) before treatment with antimycin A, rotenone, or their respective vehicles in phenol-free F-12K media. Untreated cells were used as a negative control, and *tert*-butyl hydroperoxide (T-BHP; Sigma, catalog no. 458139) was used as a positive control. After a 4-h incubation period with antimycin A or rotenone, cells were analyzed on a fluorescent plate reader (Biotek Synergy HTX; Agilent Technologies, Inc., Santa Clara, CA) at excitation (Ex) of 485/20 nm and emission (Em) of 528/20 nm. Cell treatments were assayed in duplicate, and fluorescence intensity was averaged from five independent experiments. Data presented are as fluorescence intensity after background (blank wells with media only) subtraction.

### Flow Cytometry

A cell-based flow cytometry assay kit (Abcam, catalog no. ab176749) was used to simultaneously detect apoptotic, necrotic, and viable BeWo cells after treatment with antimycin A or rotenone. For these experiments, BeWo cells were plated onto 60-mm polystyrene dishes and grown until 80% confluent. Cells were treated with antimycin A (10, 50, 100, 320 µM) or rotenone (0.8, 5, 10, 25, 50 µM) as described above. Cell culture supernatants (floating cells) were collected. Adherent cells were collected with 0.25% trypsin-EDTA for 3–5 min at 37°C. The trypsin reaction was quenched with growth medium, and the adherent cell suspension was added to the previously collected cell culture supernatant (floating cells). The cell suspension containing adherent cells and cell culture supernatant (floating cells) were centrifuged for 5 min at 400 *g* at 4°C. Pellets were rinsed with ice-cold PBS and centrifuged again for 5 min at 200 *g* at 4°C. Cells were resuspended in 200 μL of assay buffer, according to the manufacturer’s instructions, and then stained for 30 min at room temperature with 2 μL of 7AAD (Ex/Em: 546/647 nm), Apopxin green (Ex/Em: 490/525 nm), and cyto-calcein violet (Ex/Em: 405/450 nm) to detect necrosis, apoptosis, and viability, respectively. Dye concentration was determined after preliminary titration experiments. After staining, samples were read with an LSR II FACS/flow cytometer (BD Biosciences, San Jose, CA) and analyzed with FlowJo flow cytometry software (v.10; Becton, Dickinson & Company, Ashland, OR). Viable cells were defined as cells stained positive for cyto-calcein but negative for 7AAD and Apopxin green (CC^+^, 7AAD^−^, Apo^−^). Apoptotic cells were defined as cells stained positive for Apopxin green (Apo^+^, 7AAD^+^ and Apo^+^, 7AAD^−^), whereas necrotic cells were defined as those cells stained positive for 7AAD and negative for Apopxin green (7AAD^+^, Apo^−^). We identified two populations of cells. Each gating strategy was applied separately to each population (Supplemental Fig. S2, *A* and *C*, see https://doi.org/10.6084/m9.figshare.25091618). We calculated the sum of stained cells from *population 1* and *population 2* then divided by the total cell count from both populations to determine the percentage of viability, apoptosis, and necrosis (Supplemental Fig. S2, *B*, *D*, and *E*).

### Western Blots

To further understand mechanisms associated with the release of mtDNA, we focused on experiments with antimycin A. BeWo cells were seeded into six-well plates at a density of 100,000 cells/mL and grown until 80% confluent. Trophoblast cells were treated with antimycin A (10, 100, or 320 μM) or vehicle for 4 h. Culture medium was removed, and wells were rinsed with Dulbecco’s phosphate-buffered saline (DPBS). Protein was then extracted with lysis buffer containing cOmplete Lysis-M with protease inhibitor cocktail tablets, and protein concentrations were determined with Pierce BCA Protein Assay. Samples were denatured with β-mercaptoethanol and heated to 100°C for 5 min. Equal amounts of protein (20 μg) were loaded onto 10% or 15% SDS-PAGE gels for 3 h at 100 V. Proteins were transferred for 90 min to nitrocellulose or polyvinylidene difluoride (PVDF) membranes. Afterwards, membranes were blocked for 1 h with 3% bovine serum albumin (BSA) or with 5% nonfat dry milk [for light chain 3 (LC3)A/B] as described by Lima et al. (22). Primary antibodies were dissolved with 3% BSA in Tris-buffered saline (TBST), and membranes were incubated overnight at 4°C with Sequestosome-1 (SQSTM1)/p62 (Cell Signaling, catalog no. 88588, 1:1,000, RRID:AB_2800125). The primary LC3 A/B (Cell Signaling, catalog 4108S, 1:1,000, RRID:AB_2137703) was dissolved with 3% nonfat dry milk. Membranes were washed with TBST and then incubated with secondary antibodies [anti-rabbit IgG (H + L) DyLight 680 conjugate (LI-COR Biosciences, catalog no. 925-68071, 1:5,000, RRID:AB_2721181), anti-mouse IgG (H + L) DyLight 800 conjugate (LI-COR Biosciences, catalog no. 926-32210, 1:10,000, RRID:AB_621842), and rabbit conjugated to horseradish peroxidase (HRP) (Cell Signaling, catalog no. 7074, 1:4,000, RRID:AB_20992233)] for 1 h at room temperature. Data were acquired with Odyssey CLx LI-COR (LI-COR Biosciences) and analyzed with Image Studio v. 5.2 (LI-COR Biosciences). For LC3 A/B, data were obtained with the SuperSignal West Dura Extended Duration Substrate system (ThermoFisher Scientific) and the Azure 300 chemiluminescence detection system (Biosystem, United States), and band intensities were determined with ImageJ software 13.0.6 (National Institutes of Health). Values were normalized to total protein with Ponceau staining. Representative immunoblots are illustrated in Supplemental Fig. S3 (see https://doi.org/10.6084/m9.figshare.25091624).

### Statistical Analysis

Linear regression models were estimated to examine the association between each drug concentration (rotenone or antimycin A) and DNA quantities (membrane-bound and non-membrane-bound mtDNA and nuclear DNA). Analyses were stratified by nuclear DNA (B2M) or mtDNA (MinArc) and adjusted for batch effects by including batch as a covariate. A DNA quantity value was considered as an outlier if it was 1.5 times interquartile range above the third quartile or below the first quartile and was then removed from analyses. Partial (Cohen’s) f^2^ was calculated to measure effect sizes. A partial f^2^ of 0.02, 0.15, and 0.35 represents a small, medium, and large effect, respectively (23).

One-way ANOVA with Dunnett’s multiple comparison test (for parametric data) or Kruskal–Wallis with Dunn’s multiple comparison test (for nonparametric data) was used to examine the effect of treatment on ROS activity (DCFD data) and cell death (flow cytometry data). Unpaired *t* tests were used for group comparisons in EV-BeWo mtDNA content. Normality test (Shapiro–Wilk test), outlier removal (ROUT), and univariate analysis were performed with Prism (version 8; GraphPad, San Diego, CA). Data are presented as means ± SD (unless otherwise specified), and individual data points are included in figures. The significance level was set at α = 0.05 for all comparisons, and exact *P* values are presented.

## RESULTS

### Oxidative Stress and mtDNA Release from Trophoblast Cells

**Figure 2.**
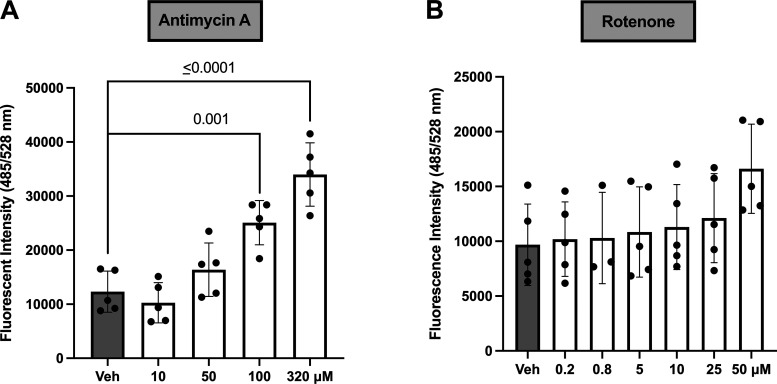
Cytosolic reactive oxygen species (ROS) levels in BeWo trophoblast cells after exposure to antimycin A or rotenone. A 2′,7′-dichlorodihydrofluorescein diacetate (DCFDA) assay was performed to determine ROS levels in BeWo cells treated with antimycin A (*n* = 5/concentration; *A*) or rotenone (*n* = 3–5/concentration; *B*). Significance was determined by 1-way ANOVA with Dunnett’s post hoc analysis. All values presented as means ± SD; *n* indicates independent experiments. Veh, vehicle.

Cytosolic ROS activity was increased in response to antimycin A (1-way ANOVA, *P* < 0.0001; Fig. 2*A*) but not in response to rotenone (1-way ANOVA, *P* = 1.79; Fig. 2*B*).

**Figure 3.**
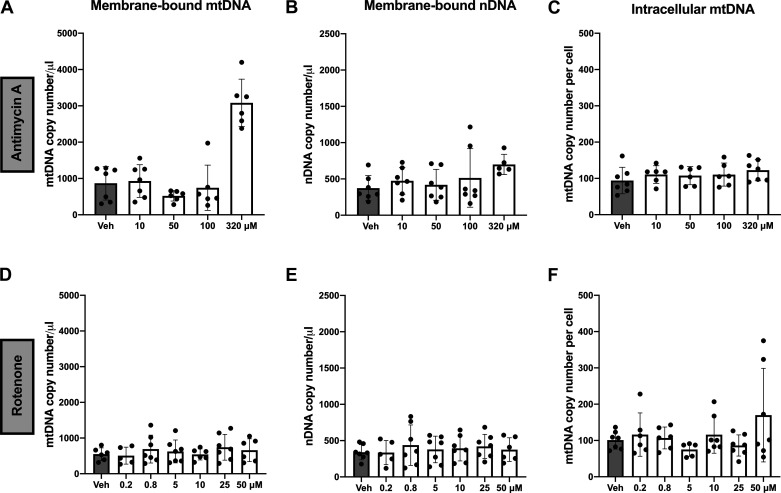
Membrane-bound extracellular mitochondrial (mt)DNA and nuclear (n)DNA and intracellular mtDNA content in BeWo trophoblast cells in response to antimycin A and rotenone. *A–C*: antimycin A increased the release of membrane-bound mtDNA (*P* < 0.0001; *A*) and nDNA (*P* = 0.024; *B*), but there was no effect on intracellular mtDNA copy number (*P* = 0.62; *C*). *D–F*: rotenone did not affect the release of membrane-bound mtDNA (*P* = 0.43; *D*), nDNA (*P* = 0.82; *E*), or intracellular mtDNA (*P* = 0.054; *F*). All values presented as means ± SD; *n* = 5–7 independent observations. Significance was determined with a linear regression model (drug concentration vs. DNA quantities) stratified by nDNA (B2M) or mtDNA (MinArc) and adjusted for batch effects. Veh, vehicle.

There was a positive association between treatment with antimycin A and release of membrane-bound mtDNA (linear regression, *P* < 0.0001, partial f^2^ = 1.73; Fig. 3*A*) and nuclear DNA (linear regression, *P* = 0.024, partial f^2^ = 0.14; Fig. 3*B*) in cell culture supernatants. Antimycin A treatment was not associated with intracellular mtDNA copy number (linear regression, *P* = 0.62, partial f^2^ = 0.006; Fig. 3*C*). There was no association between rotenone treatment and membrane-bound mtDNA (linear regression, *P* = 0.43, partial f^2^ = 0.014; Fig. 3*D*), nuclear DNA (linear regression, *P* = 0.82, partial f^2^ = 0.001; Fig. 3*E*), or intracellular mtDNA copy number (linear regression, *P* = 0.054, partial f^2^ = 0.0849; Fig. 3*F*).

**Figure 4.**
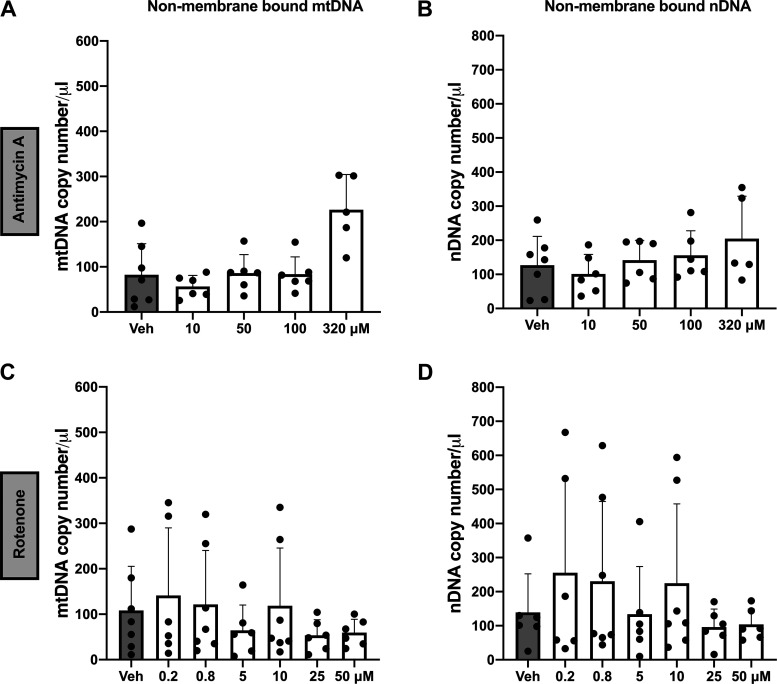
Non-membrane-bound extracellular mitochondrial (mt)DNA and nuclear (n)DNA in BeWo trophoblast cells in response to antimycin A and rotenone. *A* and *B*: antimycin A increased the release of non-membrane-bound mtDNA (*P* < 0.001; *A*) but not nDNA (*P* = 0.051; *B*). *C* and *D*: rotenone did not affect the release of non-membrane-bound mtDNA (*P* = 0.180; *C*) or nDNA (*P* = 0.115; *D*). All values presented as means ± SD; *n* = 5–7 independent observations. Significance was determined with a linear regression model (drug concentration vs. DNA quantities) stratified by nDNA (B2M) or mtDNA (MinArc) and adjusted for batch effects. Veh, vehicle.

Treatment with antimycin A was positively associated with non-membrane-bound mtDNA in cell culture supernatants (linear regression, *P* < 0.001, partial f^2^ = 0.84; Fig. 4*A*), but it was not associated with non-membrane-bound nuclear DNA (linear regression, *P* = 0.051, partial f^2^ = 0.113; Fig. 4*B*). Exposure to rotenone was not associated with non-membrane-bound mtDNA (linear regression, *P* = 0.180, partial f^2^ = 0.039; Fig. 4*C*) or nuclear DNA (linear regression, *P* = 0.115, partial f^2^ = 0.057; Fig. 4*D*) in BeWo cell culture supernatants.

**Figure 5.**
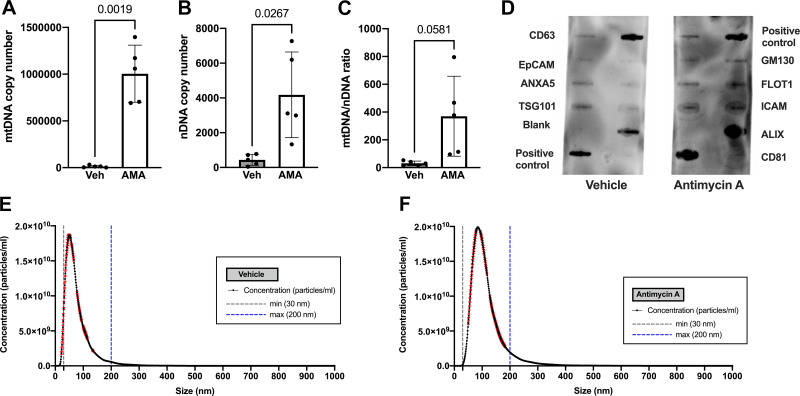
Mitochondrial (mt)DNA) and nuclear (n)DNA content in BeWo-derived extracellular vesicles (EV-BeWo) in response to oxidative stress. *A–C*: EV-BeWo released in response to antimycin A (AMA) had greater content of mtDNA (*A*), nDNA (*B*), and mtDNA-to-nDNA ratio (*C*). All values presented as means ± SD; *n* = 5 independent observations/treatment. Significance was determined by unpaired *t* tests. Veh, vehicle. *D*: semiquantitative ExoCheck Exosome Antibody Array was exposed to 50 μg of exosomal proteins isolated from pooled BeWo cell supernatant with ExoQuick. Positive exosomal markers include CD63, CD81, ALIX [programmed cell death 6 interacting protein (PDCD6IP)], FLOT-1 (flotillin-1), ICAM-1 (intercellular adhesion molecule 1), EpCam (epithelial cell adhesion molecule), ANX5 (annexin 5), and TSG101 (tumor susceptibility gene 101). A labeled positive control for horseradish peroxidase (HRP) detection and a blank spot as a background control have been included. *E* and *F*: nanoparticle tracking analysis was used to determine size distribution (nm) of EVs from BeWo cells treated with vehicle (*E*) and antimycin A (*F*). Each graph depicts the average of 3 readings for each group. The threshold lines at 30 nm (gray dotted line) and 200 nm (blue dotted line) denote the size range of small EVs. Red error bars indicate ±SE.

Antimycin A increased the membrane-bound mtDNA-to-nuclear DNA ratio (1-way ANOVA, *P* = 0.016; Supplemental Fig. S4*A*, see https://doi.org/10.6084/m9.figshare.25091642) and the non-membrane-bound mtDNA-to-nuclear DNA ratio (1-way ANOVA, *P* = 0.004; Supplemental Fig. S4*B*). Rotenone did not affect the membrane-bound (Kruskal–Wallis, *P* = 0.643) and non-membrane-bound (1-way ANOVA, *P* > 0.99) mtDNA-to-nuclear DNA ratio (Supplemental Fig. S4, *C* and *D*). To determine whether increases in membrane-bound DNA reflect an increase in vesicle-contained DNA content, we isolated EV-BeWo from supernatants of cells treated with antimycin A (320 µM) or vehicle. We chose this concentration of antimycin A because we previously observed an increase in membrane-bound mtDNA in response to this concentration but not to other concentrations. Antimycin A increased EV-BeWo-contained mtDNA content (unpaired *t* test, *P* = 0.0019; Fig. 5*A*), nuclear DNA (unpaired *t* test, *P* = 0.0267; Fig. 5*B*), and the mtDNA-to-nuclear DNA ratio (unpaired *t* test, *P* = 0.0581; Fig. 5*C*) compared with vehicle. Semiquantitative ExoCheck Exosome Antibody Array showed that the isolated vesicles expressed exosome-associated protein markers (Fig. 5*D*). Finite track length analysis of EV-BeWo revealed a size distribution of 117.1 ± 0.9 nm (antimycin A; Fig. 5*F*) and 75.2 ± 1.0 nm (vehicle; Fig. 5*E*).

### Oxidative Stress, Cell Death, and Autophagy in Trophoblast Cells

**Figure 6.**
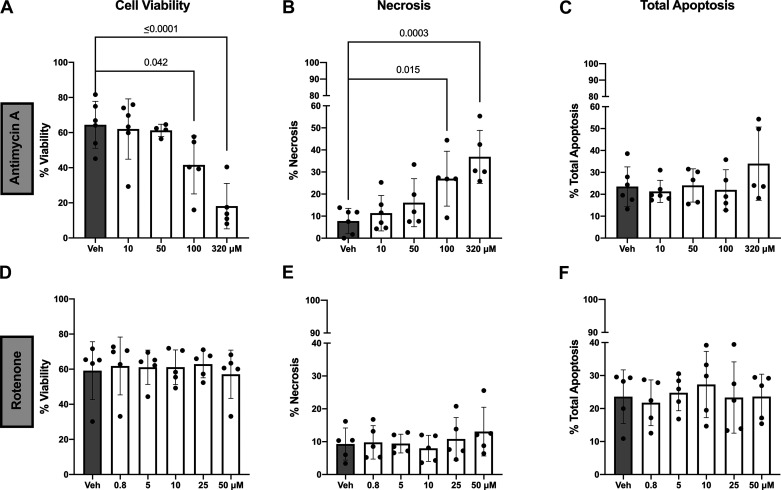
Effect of antimycin A and rotenone on cell viability and death in BeWo trophoblast cells. *A–C*: antimycin A (*n* = 5) decreased cell viability (*A*), increased necrosis (*B*), and did not affect apoptosis (*C*). *D–F*: rotenone (*n* = 3–5) did not affect cell viability (*D*), necrosis (*E*), or apoptosis (*F*). Data were analyzed by 1-way ANOVA with Dunnett’s multiple comparison test (for parametric data) or Kruskal–Wallis with Dunn’s multiple comparison test (for nonparametric). All cells are presented as a percentage of total cells. All values presented as means ± SD; *n* indicates independent observations/drug concentration. Veh, vehicle.

Antimycin A decreased cell viability (1-way ANOVA, *P* < 0.0001; Fig. 6*A*), increased necrosis (1-way ANOVA, *P* = 0.0004; Fig. 6*B*), but did not affect apoptosis (Kruskal–Wallis, *P* = 0.65; Fig. 6*C*). Rotenone had no effect on cell viability, necrosis, or apoptosis (*P* ≥ 0.75; Fig. 6, *D*–*F*). Supplemental Fig. S2 shows the gating strategy for the flow cytometry data analysis.

**Figure 7.**
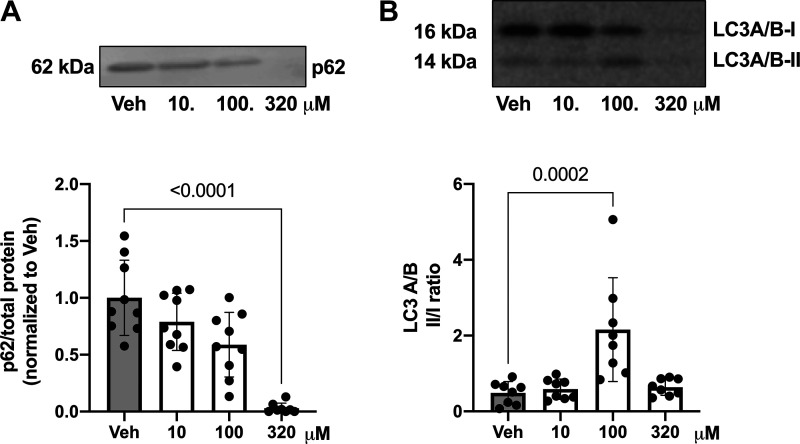
BeWo trophoblast cell protein content of autophagy markers p62 and LC3 after exposure to antimycin A. Antimycin A decreased protein content of p62 (*A*) and increased LC3 A/B II-to-I ratio (*B*) in BeWo cells. Representative immunoblots are presented above the graphs. Band intensity for p62 is normalized to total protein (Ponceau S stain). p62 summary data are presented relative to the mean of vehicle controls (Veh; mean value was set to 1). Values are shown as means ± SD. *n* = 7–9 observations/drug concentration. Data were analyzed with 1-way ANOVA (and Dunnett’s multiple comparison test) or Kruskal–Wallis (and Dunn’s multiple comparison test).

Antimycin A reduced protein content of p62 (1-way ANOVA, *P* < 0.0001; Fig. 7*A*) and increased protein content of LC3 A/B II-to-I ratio (1-way ANOVA, *P* = 0.0040; Fig. 7*B*).

In additional experiments, BeWo cells were treated with *tert*-butyl hydroperoxide (T-BHP, 200 µM) for 24 h. Our results indicate that treatment of BeWo cells with T-BHP did not reduce cell viability (Supplemental Fig. S5*A*, see https://doi.org/10.6084/m9.figshare.25648815) and did not induce apoptosis (Supplemental Fig. S5*B*) or necrosis (Supplemental Fig. S5*C*) as assessed by flow cytometry. Furthermore, exposure to T-BHP for 24 h induced an increase in ROS (DCFDA assay; Supplemental Fig. S5*D*) and did not increase release of mtDNA [Supplemental Fig. S5, *E* (pg/μL) and *F* (copies/cell)]. These observations suggest that nonlethal ROS does not induce the release of mtDNA in the extracellular space in BeWo cells.

## DISCUSSION

The main findings of this study are that *1*) an increase in ROS triggers release of mtDNA in BeWo cells, a well-established in vitro model of human trophoblast cells, and *2*) mtDNA is released from BeWo trophoblast cells in various biological forms, namely membrane bound, non-membrane bound, and vesicle bound. Furthermore, our results suggest that autophagy activation and cell necrosis, but not cell apoptosis, are involved in ROS-mediated mtDNA release from BeWo cells. This is the first study to interrogate the ability of trophoblast cells to release mtDNA in response to ROS and to identify mechanisms of release and biological forms of mtDNA that derives from trophoblast cells.

ccf-mtDNA is used as an accessible blood-based surrogate of cellular stress and inflammation to assess disease progression and severity (12), survival rates (13), and disease prognosis (14) in nonobstetric pathologies. Establishing the cellular origin of ccf-mtDNA in the circulation may provide information about organ function, mechanisms of release, and cellular signaling (11). Various types of nonplacental cells have the ability to release mtDNA, including platelets (24), leukocytes (25), endothelial cells (26), and immortalized cell lines (26–31). We and others have shown that maternal ccf-mtDNA concentrations are dysregulated in obstetric complications such as preeclampsia and intrauterine growth restriction (7–10). The source of maternal ccf-mtDNA in these studies was not investigated, and it was hypothesized that it may originate from the placenta and function as an indicator of placental cell turnover and tissue function (7–10, 32). Indeed, recent studies confirm that first-trimester human placental explants can release mtDNA (33). Similarly, our present findings demonstrate that BeWo cells, an in vitro model of trophoblast cells, release mtDNA constitutively and this release is exaggerated when the cells are exposed to stress.

An increase in ROS is a known trigger of mtDNA release in nonplacental tissues (16, 17). Our data are the first to show that an increase in ROS triggers the release of mtDNA from BeWo trophoblast cells as well. Placental ROS is highly relevant to obstetric complications because it is associated with placental ischemia, mitochondrial dysfunction, and inflammation, all of which contribute to adverse perinatal outcomes (2, 34, 35). Exposure to pharmacological inhibition of the electron transport chain, either with rotenone or antimycin A (inhibitors of complexes I and III, respectively) or other cytotoxic agents such as hydrogen peroxide and low levels of oxygen, is often used to induce ROS production in in vitro experimental setups (36). Findings from these studies suggest an association between increased ROS production and cell death in placental (19, 37, 38) and nonplacental (39, 40) cells. Here, we posit that this relationship also mediates the release of mtDNA into the extracellular space in trophoblast cell cultures. Our data show that an increase in trophoblast cell ROS triggers necrosis, but not apoptotic cell death, and causes a dose-dependent increase in the release of mtDNA. In the placental cell lines BeWo, JEG-3, and Swan-71, exposure to rotenone or antimycin A led to a dose-dependent increase in ROS and also led to apoptotic cell death in rotenone-treated cells after 4 h (19). ROS-mediated increase in apoptotic cell death has also been reported in response to hypoxia or hydrogen peroxide (37, 38). Nonapoptotic cell death and release of mtDNA, however, were not determined in any of these previous investigations. When we treated cells with T-BHP (200 µM), we observed an increase in ROS but no increases in cell death or mtDNA release. Furuta et al. (41) recently showed that treatment with hydrogen peroxide impaired viability and growth of BeWo cells only at concentrations > 500 µM. In contrast, other trophoblast cell lines (i.e., HchEpC1b-mSt, HTR8-mSt, and TCL1) showed growth inhibition after treatment with 12.5 µM hydrogen peroxide. These data further support the notion that ROS-associated cell death leads to mtDNA release from trophoblast cells, whereas nonlethal ROS does not induce mtDNA release.

BeWo cells spontaneously form syncytia, and the level of syncytialization could affect the sensitivity of the cells to ROS-mediated apoptosis (42). Hence, differences in levels of syncytialization before treatment between studies could explain differential outcomes in cell death (42, 43). The contribution of spontaneous syncytialization, however, is rather unlikely since the rates of spontaneous syncytialization in BeWo cells not treated with a cAMP analog are low (∼5–15% fusion in undifferentiated cells) (44, 45). Furthermore, disparate effects of rotenone and antimycin A on cell differentiation could explain an increased tolerance to rotenone in BeWo cells as indicated by reduced levels of ROS production and higher viability compared with antimycin A (46, 47). Overall, our findings suggest that treatment type and ROS severity may contribute to the differences observed in cell death outcomes and mtDNA release in response to rotenone or antimycin A in BeWo cells (48).

ccf-mtDNA can be released in multiple biological forms under physiological and pathological conditions (49). In line with these findings, we demonstrate that mtDNA is released from trophoblast cells constitutively and in response to stress in both membrane-bound and non-membrane-bound forms. These findings agree with our previous reports in human pregnancies complicated with preeclampsia (10). It has been suggested that the membrane-bound form of mtDNA may be protected from degradation and play a role in signaling mechanisms primarily through extracellular vesicles (11). During pregnancy, the placenta releases vesicles such as syncytial nuclear aggregates, macrovesicles, nanovesicles, and exosomes (50). Studies have shown that pregnancies with obstetric complications have differences in cargo and size of vesicles released from trophoblast cells (33). To investigate whether mtDNA from trophoblast cells is sequestered into extracellular vesicles when released from trophoblast cells, we quantified mtDNA copy number in EV-BeWo and found an increase in mtDNA levels stored in EV-BeWo exposed to ROS. This finding indicates that trophoblast cells release EVs enriched in mtDNA in response to ROS and cell death. However, we do not exclude the possibility that mtDNA is also contained in other vesicular structures derived from these cells such as liposomes or apoptotic bodies. The characterization of EVs in our study (i.e., NTA) indicates, however, that there are not any apoptotic bodies present in our samples, as there was no additional peak and no signal showing at sizes > 500 nm (51). Our data establish the foundation for future investigations to use markers of EV-containing mtDNA to establish cellular origin of ccf-mtDNA in an in vivo model.

Previous research implicated the role of autophagy impairment in placental pathology associated with ROS (52–58). Studies in trophoblast cells demonstrated an activation of autophagy after exposure to hypoxia (56, 57) or pharmacological inducers of ROS (37), with increased expression of LC3 and decreased p62 expression. Changes in LC3 expression indicate changes in autophagosome formation or degradation, and changes in p62, which is a cargo loading protein, reflect changes in autophagic flux (59). In normal autophagy, an increase in LC3 is accompanied by a reduction in p62. In this study we demonstrated that ROS increased autophagy as suggested by an increase in LC3 and a reduction in p62 in response to 100 µM of antimycin A, which is a concentration that induced oxidative stress in BeWo cells. It is possible that the lower concentrations of antimycin A have no effect on autophagy and thus we do not see a significant change in LC3 (or p62). It is noteworthy that these lower concentrations do not induce oxidative stress or cell death. In concentrations higher than 100 µM, the lack of a concomitant decrease in p62 with an increase in LC3 may reflect a disruption in the autophagic process, which then contributes to high levels of cell necrosis. We hypothesize that ROS mediates the balance between autophagy and cell death pathways, with autophagy functioning as a cytoprotective mechanism until high levels of ROS initiate cell death and release of mtDNA (54, 56, 60). Additional studies are needed to investigate the role of autophagy in mtDNA release and its role in placental pathology. Furthermore, more studies are needed to understand the effects of ROS on autophagosome formation and degradation in trophoblast cells. Moreover, other mechanisms, including mitophagy (56, 61, 62) and antioxidant production (34, 60), may also be implicated as cytoprotective mechanisms in trophoblast cell responses to ROS, and their relationship to mtDNA release warrants further investigation (34, 63).

### Conclusions, Limitations, and Future Directions

The main objective of the present study was to establish an association between oxidative stress, which is a common feature of placental pathology in obstetric complications, and mtDNA release mechanisms. We tested our hypothesis using a commonly used in vitro model of trophoblast cells, the BeWo choriocarcinoma monolayer cell line. Proteomics analysis has shown that the protein profile of BeWo cells resembles that of villous trophoblasts (64). This cell line has been widely used since the 1980s as a surrogate of villous trophoblast cells (65). Although primary trophoblast cells would be an ideal choice, they can be used only for short-term experiments because of their inability to proliferate in culture (66). A limitation of using BeWo cells is that we cannot address the effects of gestational age on ROS-mediated release of mtDNA or the interaction of trophoblast cells with other cell types (i.e., endothelial cells), and thus future experiments in placental explants or organoids may be necessary to validate our findings. It is noteworthy that BeWo cells can spontaneously syncytialize, although their basal fusion rate, in the absence of an inducer, is relatively low compared with other choriocarcinoma cell lines (66). Nevertheless, because of their ability to spontaneously fuse to form syncytia, BeWo cells may express both cytotrophoblast and syncytiotrophoblast markers. In our flow cytometry experiments, we noted the presence of two distinct cell populations of BeWo cells. This heterogeneity may reflect syncytialized and nonsyncytialized cells; however, we did not specifically characterize these. Similar data have been previously reported by other investigators (19). Future studies should examine the propensity of various trophoblast cell types to release mtDNA in response to stress using primary trophoblast cells or other in vitro models such as placental explants. In the present investigation, we measured mtDNA content in BeWo-derived EVs based on our findings that most ccf-mtDNA (10) as well as extracellular mtDNA in cell cultures (present study) is membrane bound. In the EV characterization data, we noted expression of Golgi matrix protein 130 (GM130), a marker of the Golgi apparatus. This indicates that there could be apoptotic bodies present in samples; however, as mentioned above, the NTA data do not show a signal at >500+ nm size (67). This finding might also reflect contamination from nonexosomal proteins due to coprecipitation as a result of the polymer-based precipitation technique utilized to isolate exosomes (68). Using qPCR-based methods for mtDNA quantification, we cannot be certain that the targets of the qPCR reaction are of mtDNA origin because of the known presence of mtDNA-based sequences in the nuclear genome (known as NUMTs). However, the MinArc mtDNA primers used here are designed with stringent alignment parameters to maximize specificity; this combined with *1*) the static nature of nuclear DNA copy number in diploid cells and *2*) the amplified nature of mtDNA reduces the risk of artificially inflated mtDNA quantifications due to off-target NUMT amplification.

In conclusion, this is the first study to demonstrate that BeWo cells, a well-established model of trophoblast cells, release mtDNA constitutively and this release is enhanced in response to ROS. Furthermore, we established an association between ROS, mtDNA release, autophagy, and nonapoptotic cell death mechanisms. The present investigation reveals molecular targets and potential mechanisms that can be used in future investigations to validate our main outcomes in nonimmortalized trophoblast cells and to investigate the source and biomarker potential of ccf-mtDNA in in vivo preclinical models and humans.

## DATA AVAILABILITY

Data will be made available upon reasonable request.

## SUPPLEMENTAL MATERIALS

10.6084/m9.figshare.25761720 Supplemental Table S1: https://doi.org/10.6084/m9.figshare.25761720.

10.6084/m9.figshare.25761732 Supplemental Methods: https://doi.org/10.6084/m9.figshare.25761732.

10.6084/m9.figshare.25091609 Supplemental Fig. S1: https://doi.org/10.6084/m9.figshare.25091609.

10.6084/ms9.figshare.25091618 Supplemental Fig. S2: https://doi.org/10.6084/ms9.figshare.25091618.

10.6084/m9.figshare.25091624 Supplemental Fig. S3: https://doi.org/10.6084/m9.figshare.25091624.

10.6084/m9.figshare.25091642 Supplemental Fig. S4: https://doi.org/10.6084/m9.figshare.25091642.

10.6084/m9.figshare.25648815 Supplemental Fig. S5: https://doi.org/10.6084/m9.figshare.25648815.

## GRANTS

This study was supported by NIH R01 HL146562 and HL146562-S2 to S.G., AHA 18PRE33960162 and NIH T32 AG020494 to S.C.C., AHA 19TPA-34850131 to S.G., AHA 22POST-903250 to J.L.B., AHA 22PRE-900431 to J.J.G., and AHA 23PRE-1012811 to S.M.T.

## DISCLAIMERS

The content is solely the responsibility of the authors and does not necessarily represent the official views of the National Institutes of Health.

## DISCLOSURES

No conflicts of interest, financial or otherwise, are declared by the authors.

## AUTHOR CONTRIBUTIONS

J.J.G., S.C.C., and R.O. conceived and designed research; J.J.G., S.C.C., R.O., J.L.B., N.H., I.K.G., S.M.T., Z.Z., N.R.P., and S.G. performed experiments; J.J.G., S.C.C., R.O., J.L.B., Z.Z., R.L.C., N.R.P., and S.G. analyzed data; J.J.G., S.C.C., R.O., J.L.B., N.H., I.K.G., S.M.T., Z.Z., R.L.C., N.R.P., and S.G. interpreted results of experiments; J.J.G., S.C.C., R.O., J.L.B., N.H., and I.K.G. prepared figures; J.J.G. and S.C.C. drafted manuscript; J.J.G., S.C.C., R.O., J.L.B., N.H., I.K.G., S.M.T., Z.Z., R.L.C., N.R.P., and S.G. edited and revised manuscript; J.J.G., S.C.C., R.O., J.L.B., N.H., I.K.G., S.M.T., Z.Z., R.L.C., N.R.P., and S.G. approved final version of manuscript.
